# Supplementary material for: Complete Genome Sequence of Herpes Simplex Virus 2 Strain G
Source: Viruses. 2022 Mar 5;14(3):536. doi: 10.3390/v14030536 (PMC8954253; doi:10.3390/v14030536)
Supplement: Supplementary file 1 [file viruses-14-00536-s001.zip › TableS1.pdf]

Table S1 G vs MS Insertion/Deletion

strain G	G.length	MS.length	iden%	Substitution	Insertion	Deletion
RL1	771	762	96.3%	3	15	6
RL2	2418	2406	99.1%	4	12	0
UL26	1911	1917	99.6%	1	0	6
UL29	3588	3594	99.6%	8	0	6
UL30	3723	3717	99.8%	1	6	0
UL39	3426	3435	99.6%	6	0	9
UL46	2169	2166	99.2%	8	6	3
UL49	903	909	99.0%	3	0	6
RS1	4023	3996	96.0%	13	89	62
US2	882	876	99.1%	2	6	0
US8	1647	1638	99.3%	3	9	0
US12	855	909	94.1%	0	0	54
US11	489	456	96.7%	1	0	49*

* 54 bp Deletion in strain G genomic sequence cause frameshift of last two amino acids and additional 20 amino acids added at the end.
