# Supplementary material for: Complete Genome Sequence of Herpes Simplex Virus 2 Strain G
Source: Viruses. 2022 Mar 5;14(3):536. doi: 10.3390/v14030536 (PMC8954253; doi:10.3390/v14030536)
Supplement: Supplementary file 1 [file viruses-14-00536-s001.zip › TableS2.pdf]

Table S2. G vs MS Substitution Only

Gene CDS	G.length	MS.length	iden%	Substitution	Deletion	Insertion
UL1	675	675	99.9%	1	0	0
UL2	1005	1005	99.7%	3	0	0
UL4	606	606	99.8%	1	0	0
UL5	2646	2646	99.9%	3	0	0
UL6	2037	2037	100.0%	1	0	0
UL8	2259	2259	99.8%	4	0	0
UL9	2616	2616	99.9%	2	0	0
UL10	1404	1404	99.9%	2	0	0
UL12	1863	1863	99.9%	2	0	0
UL13	1557	1557	99.9%	2	0	0
UL14	660	660	99.8%	1	0	0
UL17	2109	2109	99.9%	2	0	0
UL18	957	957	99.9%	1	0	0
UL19	4125	4125	99.9%	6	0	0
UL20	669	669	99.7%	2	0	0
UL21	1599	1599	99.8%	3	0	0
UL22	2517	2517	99.7%	7	0	0
UL23	1131	1131	99.8%	2	0	0
UL24	846	846	99.9%	1	0	0
UL25	1758	1758	99.9%	2	0	0
UL26.5	990	990	99.9%	1	0	0
UL27	2706	2706	99.7%	8	0	0
UL28	2358	2358	99.4%	15	0	0
UL31	918	918	99.8%	2	0	0
UL32	1791	1791	99.8%	3	0	0
UL33	393	393	99.7%	1	0	0
UL34	831	831	99.8%	2	0	0
UL36	9300	9300	99.9%	9	0	0
UL37	3345	3345	99.9%	2	0	0
UL38	1401	1401	99.7%	4	0	0
UL40	1014	1014	99.7%	3	0	0
UL41	1479	1479	99.7%	5	0	0
UL42	1413	1413	99.8%	3	0	0
UL43	1245	1245	99.7%	4	0	0
UL44	1443	1443	99.8%	3	0	0
UL45	519	519	99.8%	1	0	0
UL47	2091	2091	99.8%	5	0	0
UL48	1473	1473	99.9%	2	0	0
UL50	1110	1110	99.9%	1	0	0
UL52	3201	3201	99.8%	6	0	0
UL53	1017	1017	99.5%	5	0	0
UL54	1539	1539	99.9%	1	0	0
UL56	708*	738	99.6%	3	0	0
US1	1245	1245	99.7%	4	0	0
US3	1446	1446	99.6%	6	0	0
US4	2097	2097	99.5%	11	0	0
US5	279	279	99.6%	1	0	0
US6	1182	1182	99.8%	2	0	0
US7	1119	1119	99.6%	5	0	0
US8A	441	441	99.8%	1	0	0
US9	270	270	99.6%	1	0	0
US10	261	261	99.2%	2	0	0
