# Supplementary material for: Complete Genome Sequence of Herpes Simplex Virus 2 Strain G
Source: Viruses. 2022 Mar 5;14(3):536. doi: 10.3390/v14030536 (PMC8954253; doi:10.3390/v14030536)
Supplement: Supplementary file 1 [file viruses-14-00536-s001.zip › TableS3.pdf]

Table S3 G vs HG52 Insertion/Deletion

Gene_Name	G.length	HG52.length	iden%	substitution	Deletion	Insertion
RL1	771	786	96.95%	5	0	15
RL2	2418	2505	98.48%	7	12	99
UL26	1911	1914	99.63%	4	0	3
UL27	2706	2715	99.52%	4	0	9
UL29	3588	3591	99.78%	5	0	3
UL32	1791	1797	99.55%	2	0	6
UL36	9300	9369	99.15%	11	0	69
UL39	3426	3429	98.69%	30	6	9
US2	882	876	99.21%	1	6	0
US4	2097	2100	99.48%	8	0	3
US8	1647	1638	99.27%	3	9	0
US12	855	909	93.95%	1	0	54
US11	489	456	??	0	0	49*
RS1	4023	3957	98.06%	6	69	3

* 54 bp Deletion in strain G genomic sequence cause frameshift of last two amino acids and additional 20 amino acids added at the end.
