# Supplementary material for: Complete Genome Sequence of Herpes Simplex Virus 2 Strain G
Source: Viruses. 2022 Mar 5;14(3):536. doi: 10.3390/v14030536 (PMC8954253; doi:10.3390/v14030536)
Supplement: Supplementary file 1 [file viruses-14-00536-s001.zip › TableS4.pdf]

Table S4 G vs HG52 Substitution Only

Gene_Nar	G.length	HG52.leng	iden%	sub	del	ins	ins/del
UL1	675	675	99.85%	1	0	0	0
UL2	1005	1005	99.70%	3	0	0	0
UL5	2646	2646	99.89%	3	0	0	0
UL6	2037	2037	99.95%	1	0	0	0
UL8	2259	2259	99.87%	3	0	0	0
UL9	2616	2616	99.96%	1	0	0	0
UL10	1404	1404	99.93%	1	0	0	0
UL11	291	291	98.97%	3	0	0	0
UL12	1863	1863	99.68%	6	0	0	0
UL13	1557	1557	99.81%	3	0	0	0
UL14	660	660	99.85%	1	0	0	0
UL17	2109	2109	99.86%	3	0	0	0
UL19	4125	4125	99.88%	5	0	0	0
UL20	669	669	99.85%	1	0	0	0
UL22	2517	2517	99.92%	2	0	0	0
UL25	1758	1758	99.83%	3	0	0	0
UL26.5	990	990	99.80%	2	0	0	0
UL28	2358	2358	99.87%	3	0	0	0
UL30	3723	3723	99.97%	1	0	0	0
UL31	918	918	99.89%	1	0	0	0
UL34	831	831	99.76%	2	0	0	0
UL37	3345	3345	99.85%	5	0	0	0
UL38	1401	1401	99.86%	2	0	0	0
UL40	1014	1014	99.90%	1	0	0	0
UL41	1479	1479	99.80%	3	0	0	0
UL42	1413	1413	99.93%	1	0	0	0
UL43	1245	1245	99.92%	1	0	0	0
UL44	1443	1443	99.86%	2	0	0	0
UL47	2091	2091	99.86%	3	0	0	0
UL48	1473	1473	99.86%	2	0	0	0
UL49	903	903	99.89%	1	0	0	0
UL49A	264	264	99.62%	1	0	0	0
UL51	735	735	99.86%	1	0	0	0
UL52	3201	3201	99.78%	7	0	0	0
UL53	1017	1017	99.51%	5	0	0	0
UL56	708	708	99.72%	2	0	0	0
US1	1245	1245	99.68%	4	0	0	0
US3	1446	1446	99.79%	3	0	0	0
US5	279	279	99.64%	1	0	0	0
US6	1182	1182	99.83%	2	0	0	0
US7	1119	1119	99.82%	2	0	0	0
US8A	441	441	99.77%	1	0	0	0
US9	270	270	99.26%	2	0	0	0
US10	261	261	99.62%	1	0	0	0
