# Supplementary material for: Complete Genome Sequence of Herpes Simplex Virus 2 Strain G
Source: Viruses. 2022 Mar 5;14(3):536. doi: 10.3390/v14030536 (PMC8954253; doi:10.3390/v14030536)
Supplement: Supplementary file 1 [file viruses-14-00536-s001.zip › TableS5.pdf]

Table S5 G vs333 Insertion/Deletion

Gene	G.length	333.length	Iden%	Substitution	Insertion	Deletion
RL1	771	762	97.4%	5	6	15
RL2	2418	2445	96.8%	4	12	39
UL26	1911	1917	99.5%	4	0	6
UL27	2706	2715	99.5%	5	0	9
UL49	903	909	98.7%	6	0	6
UL29	3588	3591	99.7%	8	0	3
US2	882	876	99.2%	1	6	0
UL46	2169	2166	99.3%	9	5	2
UL39	3426	3435	99.6%	6	0	9
US11	489	456	96.7%	1	0	54*
US12	855	909	93.9%	1	0	54
UL52	3201	3204	99.6%	7	1	4
UL36	9300	9360	99.0%	7	15	75
RS1	4023	3969	98.4%	8	55	1

* 54 bp Deletion in strain G genomic sequence cause frameshift of last two amino acids and additional 20 amino acids added at the end.
