# Supplementary material for: Complete Genome Sequence of Herpes Simplex Virus 2 Strain G
Source: Viruses. 2022 Mar 5;14(3):536. doi: 10.3390/v14030536 (PMC8954253; doi:10.3390/v14030536)
Supplement: Supplementary file 1 [file viruses-14-00536-s001.zip › TableS7.pdf]

Table S7. Pairwise distance of HSV-2 strains

	HSV2_1192	HSV2_CtSF	HSV2_COH3818	HSV2_GSC-56	HSV2_CtSF-R	HSV2_2007-82	HSV2_2007-38	HSV2_SD90e	HSV2_G	HSV2_SD66	HSV2_JA2_JP	HSV2_9335-20	HSV2_7444-19	HSV2_J32715	HSV2_Bethesd	HSV2_MS	HSV2_CTSampl	HSV2_HG52	HSV2_K39924	HSV2_44-4198	HSV2_333	HSV1_17
HSV2_1192																						
HSV2_CtSF	0.002581																					
HSV2_COH3818	0.002861	0.001089																				
HSV2_GSC-56	0.002460	0.000272	0.000968																			
HSV2_CtSF-R	0.002852	0.001080	0.000122	0.000959																		
HSV2_2007-82	0.003097	0.003264	0.003543	0.003143	0.003534																	
HSV2_2007-38	0.003315	0.001831	0.002110	0.001710	0.002101	0.003998																
HSV2_SD90e	0.002966	0.001481	0.001761	0.001360	0.001752	0.003648	0.001629															
HSV2_G	0.001889	0.002530	0.002809	0.002409	0.002800	0.003046	0.003264	0.002915														
HSV2_SD66	0.002670	0.002184	0.002463	0.002063	0.002454	0.003352	0.002918	0.002568	0.002619													
HSV2_JA2_JP	0.002921	0.002967	0.003246	0.002846	0.003237	0.003603	0.003701	0.003352	0.002870	0.003056												
HSV2_9335-20	0.002418	0.002129	0.002409	0.002008	0.002400	0.003100	0.002863	0.002514	0.002367	0.002218	0.002804											
HSV2_7444-19	0.002569	0.002615	0.002894	0.002494	0.002885	0.003251	0.003349	0.002999	0.002517	0.002703	0.002680	0.002451										
HSV2_J32715	0.003113	0.003159	0.003438	0.003038	0.003430	0.003795	0.003893	0.003544	0.003062	0.003248	0.002474	0.002996	0.002872									
HSV2_Bethesd	0.002656	0.002702	0.002981	0.002581	0.002972	0.003338	0.003436	0.003086	0.002605	0.002790	0.002454	0.002538	0.002415	0.002646								
HSV2_MS	0.002835	0.002881	0.003161	0.002760	0.003152	0.003518	0.003615	0.003266	0.002784	0.002970	0.002947	0.002718	0.002215	0.003139	0.002682							
HSV2_CTSampl	0.002850	0.003017	0.003296	0.002896	0.003287	0.002871	0.003751	0.003402	0.002799	0.003106	0.003357	0.002854	0.003004	0.003549	0.003092	0.003271						
HSV2_HG52	0.002465	0.002632	0.002911	0.002511	0.002903	0.002486	0.003366	0.003017	0.002414	0.002721	0.002972	0.002469	0.002619	0.003164	0.002707	0.002886	0.000693					
HSV2_K39924	0.002848	0.003015	0.003294	0.002894	0.003285	0.000946	0.003749	0.003399	0.002797	0.003103	0.003354	0.002851	0.003002	0.003546	0.003089	0.003269	0.002622	0.002237				
HSV2_44-4198	0.002466	0.001668	0.001947	0.001547	0.001938	0.003148	0.002402	0.002052	0.002414	0.002068	0.002851	0.002014	0.002499	0.003043	0.002586	0.002765	0.002901	0.002516	0.002899			
HSV2_333	0.002618	0.000163	0.001126	0.000309	0.001117	0.003301	0.001868	0.001518	0.002567	0.002221	0.003004	0.002166	0.002652	0.003196	0.002739	0.002918	0.003054	0.002669	0.003052	0.001705		
HSV1_17	0.573340	0.573507	0.573786	0.573386	0.573777	0.572038	0.574241	0.573891	0.573289	0.573595	0.573847	0.573343	0.573494	0.574039	0.573581	0.573761	0.573114	0.572729	0.571789	0.573391	0.573544	
